# A Model of Tuberculosis Screening for Pregnant Women in Resource-Limited Settings Using Xpert MTB/RIF

**DOI:** 10.1155/2012/565049

**Published:** 2011-10-05

**Authors:** Eleanor R. Turnbull, Nzali G. Kancheya, Jennifer B. Harris, Stephanie M. Topp, German Henostroza, Stewart E. Reid

**Affiliations:** ^1^Tuberculosis Department, Centre for Infectious Disease Research in Zambia, 5977 Benakale Road, P.O. Box 34681, Northmead, Lusaka, Zambia; ^2^Schools of Medicine and Public Health, University of Alabama at Birmingham, AL 35233, USA

## Abstract

Timely diagnosis and treatment of maternal tuberculosis (TB) is important to reduce morbidity and mortality for both the mother and child, particularly in women who are coinfected with HIV. The World Health Organization (WHO) recommends the integration of TB/HIV screening into antenatal services but available diagnostic tools are slow and insensitive, resulting in delays in treatment initiation. Recently the WHO endorsed Xpert MTB/RIF, a highly sensitive, real-time PCR assay for *Mycobacterium tuberculosis* that simultaneously detects rifampicin resistance directly from sputum and provides results within 100 minutes. We propose a model for same-day TB screening and diagnosis of all pregnant women at antenatal care using Xpert MTB/RIF. Pilot studies are urgently required to evaluate strategies for the integration of TB screening into antenatal clinics using new diagnostic technologies.

## 1. Introduction

Tuberculosis (TB) is a leading cause of nonobstetric maternal death in resource-limited settings, accounting globally for approximately 700,000 deaths every year, the majority of which are in areas with high HIV prevalence [1, 2]. In South Africa, a screening study found that the prevalence of TB is 10 times greater in HIV-infected than in HIV-uninfected pregnant women [3] and Gouder et al. found TB prevalence among women attending antenatal care was 696/100,000 among HIV-infected women compared to 200/100,000 among HIV-negative women [4]. If untreated, maternal TB can lead to increased neonatal mortality, lower birth weights, prematurity [5, 6] and increased complications of pregnancy, including a four-fold increase in maternal morbidity through higher rates of abortion, postpartum hemorrhage, labor difficulties, and preeclampsia [7, 8]. Furthermore studies have demonstrated that HIV-infected pregnant women who are coinfected with TB are 2.5 times more likely to transmit HIV to their babies than women without TB [9] and their infants are 24 times more likely to have neonatal TB [10]. Under current standards in resource-limited settings there is a significant delay from the time of presentation to the diagnosis of TB, due to the low sensitivity and long turnaround time of available diagnostic tools, the need for multiple visits, and the nonspecificity of symptoms in pregnant women, particularly those who are HIV infected. In Mexico, Figueroa-Damian found maternal morbidity, neonatal mortality, and extreme prematurity all to be significantly higher among pregnant women with TB who started treatment late in pregnancy (25–36 weeks of gestation), whilst those treated early had minimal negative outcomes [11, 12]. In addition, rapid, early diagnosis and treatment of TB reduces transmission of TB to family members, including newborns, and the wider community [13] and reduces the number of women who are lost to followup during the lengthy TB diagnostic process. In light of these risks, it is clear that early, rapid diagnosis and treatment of both HIV and TB is critical to improve both maternal and infant outcomes [14]. The World Health Organization (WHO) recommends the integration of TB/HIV care into antenatal services and TB screening of all pregnant women in high HIV prevalence areas [15].

## 2. TB Diagnosis in Pregnant Women

To date the implementation of WHO antenatal TB screening recommendations has been limited in resource poor countries due in part to significant financial and logistical constraints [16]. Clinicians are reluctant to use chest radiography as part of the TB diagnostic workup process and sputum smear microscopy using Ziehl-Neelsen (Z-N) stain has been demonstrated to have low sensitivity, especially in HIV-infected women. The WHO estimates that Z-N microscopy detects only 58% of pulmonary TB cases in HIV-infected individuals [17]. Sensitivities even lower than this have been reported; in a South African study, eight of 370 HIV-infected pregnant women were diagnosed with culture-confirmed TB but all were smear negative [18]. Despite being the diagnostic reference, standard TB culture is not accessible in most resource-limited settings due to its complex laboratory requirements. A potential alternative is the recently recommended Xpert MTB/RIF, a real-time PCR assay for *Mycobacterium tuberculosis *that simultaneously detects rifampicin resistance directly from sputum and provides results within 100 minutes [19]. Xpert MTB/RIF has been recommended for use up to the subdistrict level, especially in settings where rapid access to appropriate treatment and care is required [20]. Results from prospective demonstration studies involving 6,648 individuals found the sensitivity of a single, direct Xpert MTB/RIF test in culture-positive cases was 90.3% (99.0% in smear-positive sputa and 76.9% in smear-negative sputa), rifampicin resistance was detected with 94.4% sensitivity and 98.3% specificity and performance was not significantly affected by HIV status [21, 22]. Operationally Xpert MTB/RIF technology has shown to be robust in various locales and can be used outside laboratory settings by lay staff with minimal training [21]. These characteristics suggest that Xpert MTB/RIF may have the potential to play a significant role in the diagnosis of TB in antenatal settings in low-resource settings with generalized HIV epidemics. 

Using the example of Zambia, there are approximately 5000 women who present monthly for antenatal care at the 25 government primary healthcare clinics in the capital, Lusaka. In these health clinics the mean gestational age of women at their first antenatal visit is 22 weeks and almost half (48%) come for only one visit [23]. Over 90% of expectant women agree to HIV testing during antenatal care and approximately 22% are HIV infected. Despite well-developed HIV testing programs in Maternal Child Healthcare (MCH) departments, there is no provision for systematic TB screening and limited data available within Zambia to guide best practice. Currently, pregnant woman presenting to MCH with TB symptoms are referred to the outpatient department (where there are often long queues) for further consultation, sputum collection, and antibiotic trials. Often multiple visits are required over several days to weeks prior to receiving a final diagnosis and women may be lost to followup before the diagnostic process is complete. 

While there is no data on the diagnostic delay between initial presentation and TB diagnosis in pregnant women, an evaluation of the 2007 WHO guidelines found that median time to TB treatment initiation was between 3–17 days for smear-negative pulmonary TB [24] and a review of records at one Lusaka HIV clinic found the average time from first presentation to pulmonary TB diagnosis to be 29 days (personal communication, S. Trollip). Due to more complicated referral systems, this delay may be even greater in MCH clinics The combination of insensitive tools and lengthy diagnostic delays highlights the urgent need to integrate TB screening into antenatal care and to pilot faster, more sensitive diagnostic tools. Introduction of the Xpert MTB/RIF technology, which provides same-day TB diagnosis with high sensitivity, could potentially allow pregnant women to be screened and started on TB treatment the same day as their antenatal visit. Based on current practice this would represent a reduction of up to 3 weeks in time to TB treatment initiation, critically given that most women present late in pregnancy. 

## 3. TB Screening Model in Antenatal Clinics

Screening pregnant women for TB in MCH makes sense both clinically and logistically as even in low-resource settings a majority of women access health care during pregnancy at least once [25]. Rather than strengthening the limited and separate TB diagnostic services at the clinic, integration of TB diagnostic and antenatal services would leverage existing clinic space and staff, simplify patient flow, and reduce waiting times. 

**Figure 1:**
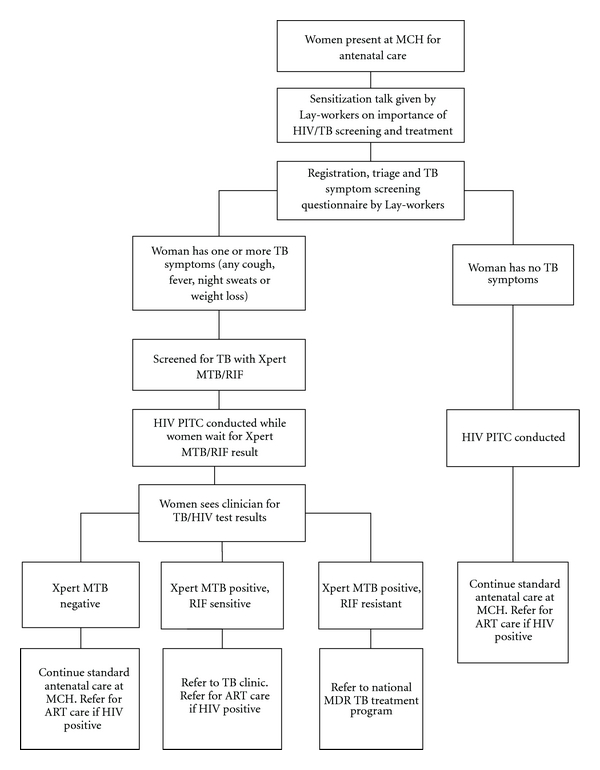
Proposed model for same-day TB screening and diagnosis of pregnant women at antenatal care using Xpert MTB/RIF (*MCH: Maternal Child Healthcare Clinic; (M)TB: (Mycobacterium) Tuberculosis; RIF: Rifampicin; PITC: Provider Initiated Testing and Counseling; ART: Antiretroviral therapy; MDR: Multidrug resistant).

We propose a model (Figure 1) to integrate TB and HIV screening, diagnosis, and treatment into existing antenatal care using Xpert MTB/RIF technology. Women present early in the morning to the antenatal clinic and while they wait for individual check-ups, group health education is given by a trained lay worker/peer educator. These health education talks provide an opportunity to deliver counseling on the TB and HIV screening services and the clinical benefits to both mother and child of TB (and HIV) screening during pregnancy. In this model, lay healthcare workers administer a simple TB symptom questionnaire that evaluates patients for key TB symptoms (any cough, fever, night sweats, or weight loss). Asymptomatic women are referred for provider-initiated counseling and testing (PITC) and continue with the standard antenatal visit where they may be considered for isoniazid prophylaxis therapy. Symptomatic (presence of *any* symptom) women are escorted to an isolated and well-ventilated area for sputum collection and afterwards for PITC. Utilizing Xpert MTB/RIF technology, sputum is analyzed in the clinic laboratory or within the MCH department, depending on space availability, security, and stable electricity supply. The clinician provides a formal assessment once TB and HIV test results are available. Women with positive TB and/or HIV test results are referred for further evaluation and, optimally, TB treatment will be initiated the same day.

## 4. Discussion

We have highlighted the need for TB screening in pregnant women and proposed a model for a point of care screening algorithm with rapid turnaround time. Variations of this model may be appropriate for different settings depending on patient flow, space, and staffing. Faster diagnosis of TB leads to earlier cotreatment, reduced transmission to community and family, and potentially improved maternal and neonatal clinical outcomes. The proposed use of Xpert MTB/RIF technology has substantial advantages in terms of sensitivity, turnaround time, and simplicity compared to existing technologies however implementation challenges exist; the instrument and storage of cartridges requires significant space, waste generated is considerably more than that with microscopy, and instruments require an uninterrupted power supply and annual validation. At present, Xpert MTB/RIF operation requires substantial expenditure, both in capital costs and consumables. In 2011, the Foundation for New Innovative Diagnostics (FIND) negotiated price for a 4-module unit (capable of running 16–20 specimens per day) is 17,000 USD and the price per cartridge is 16.86 USD (with one cartridge required per sputum tested). On the long term, prices for Xpert MTB/RIF are expected to decrease as global testing volumes increase [20]. Further work is required to examine its cost-effectiveness in various resource-limited settings. In addition, Xpert MTB/RIF technology relies on the ability of a patient to produce sputum which is problematic for those without pulmonary symptoms. Future pilots could also consider other less expensive nonsputum-based diagnostic tools that are currently under evaluation, such as a urine lateral flow test for simple and rapid lipoarabinomannan (LAM) detection [26] or simplified nucleic acid amplification tests like the loop-mediated isothermal amplification (LAMP) assay [27], after their endorsement by the WHO. 

The possible clinical benefits of an integrated system are apparent and acceptance of screening is potentially high as pregnant women generally have increased health seeking behavior to protect their unborn child; however it is important to recognize potential patient-related barriers to implementation. For example, Kali et al. found that 44% of HIV-infected pregnant women were unwilling to go through TB screening [18]. However, TB screening was offered *after* PITC, suggesting that women may have been reluctant to face another potential diagnosis immediately after learning that they were HIV infected and may be unwilling to wait the additional 2 hours required to obtain an Xpert MTB/RIF result. Women needing treatment for HIV may be especially reluctant to engage in TB screening for fear of the heavy pill burden associated with TB/HIV treatment, during pregnancy. Screening all women for both diseases on arrival, as proposed in this model, may increase uptake and strengthen acceptance of the need for routine HIV and TB screening in high-prevalence settings. Furthermore strong linkages to care and comprehensive sensitization at both clinic and community level is essential to “normalize” TB screening in the pregnant population and overcome reluctance to screen. 

Integration of HIV and TB screening services into MCH using similar models has been shown to be feasible and recommended in high HIV/TB prevalence settings [14, 16]. A simple screening questionnaire that assesses patients for any of the four key TB symptoms has been proven to accurately identify people in need of further diagnostic assessment in resource-constrained settings [28]. In addition the use of lay workers to conduct a symptom screening has been successfully piloted in South Africa where it added only 4–7 minutes to the visit time [18]. In Zambia lay workers/peer educators have been instrumental to the rapid scale-up of both HIV programs (conducting testing, adherence counseling, and followup of patients on treatment) [29, 30]; TB programs (conducting PITC, sputum collection, directly observed treatment strategy and facilitating referral linkage). As Xpert MTB/RIF instruments are reported to be simple enough to be run by lay workers this greatly increases the feasibility of using them in countries that have significant shortages of professional health care staff [21]. Despite this, implementation of a screening program, as we have proposed, will have a significant impact on the logistics of MCH visits; additional staff and appropriate space will be required for optimal implementation. 

There remain many unanswered questions about TB screening in the antenatal setting that need to be addressed by operations research. These include optimal linkage between MCH and TB care; adherence barriers and toxicity facing coinfected pregnant mothers in receiving TB and HIV cotreatment; effects of early TB/ART initiation on maternal and neonatal outcomes and neonatal HIV transmission; time to ART and TB initiation in coinfected pregnant women; feasibility and cost-effectiveness of Xpert MTB/RIF implementation in antenatal clinics; sensitivity and specificity of Xpert MTB/RIF in pregnant women; the role of lay workers/peer educators in integrated screening programs. Pilot programs are urgently needed to evaluate the impact of integrating TB screening strategies into antenatal clinics using new diagnostic technologies in order to reduce the burden of TB and HIV in mothers and their children.
